# Fatty Acid and Phenolic Compound Concentrations in Eight Different Monovarietal Virgin Olive Oils from Extremadura and the Relationship with Oxidative Stability

**DOI:** 10.3390/ijms17111960

**Published:** 2016-11-23

**Authors:** Alfonso Montaño, Marcos Hernández, Inmaculada Garrido, José Luís Llerena, Francisco Espinosa

**Affiliations:** 1 Agri-Food Technological Center of Extremadura-CTAEX, 06195 Badajoz, Spain; amontano@ctaex.com (A.M.); jllerena@ctaex.com (J.L.L.); 2 Aula Dei Scientific Technological Park Foundation, Av. Montañana 930, 50059 Zaragoza, Spain; marcoshsuarez@gmail.com; 3 Department of Plant Biology, Ecology and Earth Sciences, University of Extremadura, 06006 Badajoz, Spain; igarridoc@unex.es

**Keywords:** phenolic compounds, fatty acids, oxidative stability, virgin olive oil

## Abstract

Olive oils have been shown to be more resistant to oxidation than other vegetable fats, mainly due to their fatty acid (FA) profile which is rich in oleic acid and to their high content of antioxidants, principally phenols and tocopherols. This has situated virgin olive oils (VOOs) among the fats of high nutritional quality. However, it is important to stress that the oil’s commercial category (olive oil, virgin olive oil, extra-virgin olive oil), the variety of the source plant, and the extraction-conservation systems all decisively influence the concentration of these antioxidants and the oil’s shelf-life. The present work studied the fatty acid (FA) and phenolic composition and the oxidative stability (OS) of eight olive varieties grown in Extremadura (Arbequina, Cornicabra, Manzanilla Cacereña, Manzanilla de Sevilla, Morisca, Pico Limón, Picual, and Verdial de Badajoz), with the olives being harvested at different locations and dates. The Cornicabra, Picual, and Manzanilla Cacereña VOOs were found to have high oleic acid contents (>77.0%), while the VOOs of Morisca and Verdial de Badajoz had high linoleic acid contents (>14.5%). Regarding the phenol content, high values were found in the Cornicabra (633 mg·kg^−1^) and Morisca (550 mg·kg^−1^) VOOs, and low values in Arbequina (200 mg·kg^−1^). The OS was found to depend upon both the variety and the date of harvesting. It was higher in the Cornicabra and Picual oils (>55 h), and lower in those of Verdial de Badajoz (26.3 h), Arbequina (29.8 h), and Morisca (31.5 h). In relating phenols and FAs with the OS, it was observed that, while the latter, particularly the linoleic content (*R* = −0.710, *p* < 0.001, *n* = 135), constitute the most influential factors, the phenolic compounds, especially *o*-diphenols, are equally influential when the oils’ linoleic content is ≥12.5% (*R* = 0.674, *p* < 0.001, *n* = 47). The results show that VOOs’ resistance to oxidation depends not only on the FA or phenolic profile, but also on the interaction of these compounds within the same matrix.

## 1. Introduction

Olive oils (OOs) form the base of the Mediterranean diet. There are different commercial categories of OOs according to their quality: extra virgin olive oil (EVOO), obtained by purely physical means and with the physicochemical and sensorial parameters characteristic of healthy fruit; virgin olive oil (VOO), also obtained by purely physical means but whose physicochemical and/or sensorial parameters are not those of completely healthy fruit; and olive oil, a mix of lampante olive oil that has been refined (by a chemical process to remove any negative attributes) with some proportion of VOO or EVOO. The global consumption of OOs has increased by more than 30% over the last 25 years thanks to two key aspects: their nutritional properties [1] and their sensorial properties. Their nutritional properties are not only provided by their fatty acid (FA) profile and high monounsaturated/saturated fatty acid ratio (MUFA/SFA), but also by their antioxidant content, particularly that of phenols which intervene in the prevention of disease [2,3]. The FA and phenol contents also provide OOs with greater stability against rancidity and oxidative processes [4].

Oxidation in OOs is an irreversible process that begins at the moment the oil is extracted, and becomes more pronounced during storage. Oxygen, light, temperature, metals, pigments, and the polyunsaturated fatty acid (PUFA) composition are factors that can influence autoxidation in different ways, as also can the presence of antioxidants (both qualitatively and quantitatively).

Not all FAs have equal sensitivity to the generation of hydroperoxides. Frankel [5] calculated the relative reaction rates of the oleic:linoleic:linolenic methyl esters to be in the ratio 1:40–50:100, showing the increased sensitivity of the double bond hydrogen atoms to react with the oxygen and form hydroperoxides. It is important to highlight that the oxidative stability (OS) will not only be affected by the degree of unsaturation but also by the position and geometry of the double bonds. The *cis* FAs oxidize faster than their corresponding *trans* isomers, and conjugated double bonds are more reactive than non-conjugated. Another factor linked to FA stability is their status in the glyceride matrix, as the simple mixture of methyl esters is less stable than the glyceride matrix with an equal composition conformed as triglycerides. In addition, free FA presence in the oils also favours oxidative processes, since unsaturated fatty acids (UFAs) oxidize more rapidly than their corresponding methyl esters [4].

Phenols endow OOs with greater resistance to oxidation. They act as oxidation chain-breakers, reacting with the free radicals to form inactive radicals, and thus interrupting the chain of propagation. In fact, these compounds are capable of donating an electron or hydrogen atom to the free radical for its stabilization, transforming it into a phenoxyl radical through the relocation of an unpaired electron. This property is possessed by many antioxidants. Examples are the tocopherols and the carotenoids which contain a β-ionone ring, and especially the *o*-diphenols. Other phenols could possess this capacity, but it would depend on the transferability of a singlet hydrogen atom.

Phenols can also act as “scavengers”. They can capture free radicals, combining with peroxyl and alkoxyl radicals, and can chelate trace metals. This property seems to be more efficient in the decarboxymethyl (DOA) and aldehydic (AOA) forms of oleuropein aglycone compared with hydroxytyrosol [6].

It is well known that high OS in OOs comes primarily from the *o*-diphenols [4,7,8,9,10]. Bendini et al. [11] reviewed several studies which have shown that both oleuropein and its derivatives are better antioxidants than vitamin E, butylated hydroxytoluene (BHT), and other synthetic antioxidants which are approved for use in food. Some research has even shown that other phenols, such as elenolic acid, pinoresinol and acetoxypinoresinol, and aldehydic and decarboxymethyl derivatives of ligstroside (ALA and DLA), do not have antioxidant properties, and might even have pro-oxidant activity when tested with certain antioxidant activity evaluation methods [11,12].

The presence of hydrophilic phenols in OOs and their pronounced antioxidant activity can be explained by the “polar paradox” [11]. This indicates that polar antioxidants are more effective in non-polar lipids, but apolar antioxidants are more effective in polar lipid emulsions. Frankel [13] describes hydrophilic antioxidants, such as polar phenols, in an oil matrix as being oriented towards the air-oil interface, becoming more protective against oxidation than lipophilic antioxidants, such as tocopherols, which remain within the matrix.

The variety has a major influence on the FA and phenol concentration in EVOO—more than 70% for FA and up to 78% for phenols [14]. However, one must not forget that climate, especially in the new olive-growing countries [15], can affect FA composition. The phenol composition depends on parameters other than the variety and agroclimatic conditions, such as the ripeness of the fruit, extraction processes, etc. [16].

The purpose of this study was to determine how the FA composition and phenolic content of EVOOs influence their OS in the eight main varieties grown in Extremadura (Arbequina, Cornicabra, Manzanilla Cacereña, Manzanilla de Sevilla, Morisca, Pico Limón, Picual, and Verdial de Badajoz), with the olives harvested in different locations where these varieties are more prevalent and at different harvest dates.

## 2. Results and Discussion

### 2.1. Characterization of Phenolic Compounds

The phenolic compound content in the VOOs of the main olive varieties grown in Extremadura present significant differences in composition and concentration between those varieties (Table 1). The VOOs of the variety Arbequina are characterized by having low values of total phenols. This variety specifically has the lowest values of hydroxytyrosol, tyrosol, *p*-coumaric acid, DOA, DLA, AOA, and ALA of all the varieties studied. Another characteristic of its oils is that they have high values of hydroxytyrosol acetate and lignans + cinnamic acid. However, the VOOs of the variety Cornicabra have the highest mean values of total phenols (632.6 mg·kg^−1^). These high values are due to the DOA and DLA contents which contribute more than 70% of the total phenols.

The VOOs of the variety Manzanilla Cacereña are characterized by high content in phenols (468.8 mg·kg^−1^), among which are worth highlighting the high values of tyrosol and especially ALA. These latter are the highest among the different varieties of VOOs analysed. This variety, together with Verdial de Badajoz, has oils in which DLA is the majority phenol, with a concentration exceeding that of DOA. Another characteristic of these VOOs is that no ferulic acid was quantified and, together with Picual, they present the lowest mean values of lignans + cinnamic acid.

The VOOs of the Manzanilla de Sevilla variety have a high mean total phenol content (497.5 mg·kg^−1^). These oils are characterized by high AOA and DOA values. The AOA values, together with those of ferulic acid and apigenin, are the highest of the different varieties of oils studied. Vanillic acid and *p*-coumaric acid are also found in high concentrations in these oils, although they are not the highest of the varieties studied.

The VOOs of the variety Morisca have high phenol contents, with a mean value of 550.2 mg·kg^−1^. This is due to its high content of DLA and especially of DOA. They also present the highest values of hydroxytyrosol acetate and *p*-coumaric acid. However, the mean concentrations of two other secoiridoid compounds, AOA and ALA, are low, with values close to those of the varieties Arbequina and Verdial de Badajoz.

The VOOs of the variety Pico Limón present a high total phenol content, with a mean value of 406.8 mg·kg^−1^. Especially high is their content in hydroxytyrosol and lignans + cinnamic acid, being the highest among the varieties studied. However, they have low mean concentrations of DLA, and the lowest of *p*-coumaric acid and hydroxytyrosol acetate.

The VOOs of the variety Picual present a high total phenol content, with a mean value of 380.7 mg·kg^−1^. Oils of this variety are notable for a high content of AOA which is their main phenol. Their oils are also characterized by high concentrations of hydroxytyrosol, vanillic acid, and luteolin.

The VOOs of the variety Verdial de Badajoz have a high phenol content, with a mean value of 551.4 mg·kg^−1^. Among these phenols, DLA stands out as being the majority in this variety (together with Manzanilla Cacereña) in which it presents the highest values. The oils exhibit high values of lignans + cinnamic acid, close to those presented by the Pico Limón variety. They also have low AOA, ALA, and tyrosol acetate contents, with this last being undetected. In consulting the published literature, we found no data about the phenol content of the VOOs of this variety.

The method used for the phenol quantification influences the values obtained [4]. It is thus important to indicate which method was used as well as the form of expressing the results so as to be able to compare different methods and criteria of quantification. For example, the results reported by Andjelkovic et al. [17] and Ramos-Escudero et al. [18] show the great differences in phenol quantification in different varieties according to whether HPLC or Folin-Ciocalteu reagent methods are used.

However, despite the precision of the methods, chromatography equipment is not always available to the industry, and methods such as the Folin-Ciocalteu reagent [19] have also demonstrated their utility as a decision-making tool at an affordable cost for small production and/or packaging industries. Even the system for obtaining samples, whether from oil mills or obtained by means of other pilot plants such as the Abencor system, exert a critical influence on the final phenol content [3,20,21]. Therefore, all these results should be understood as orientative given the major deviations, both natural and intrinsic to the method used in each study.

The results obtained by other workers with the same varieties, cultivated in both Extremadura and other regions, largely coincide with the data presented here. Many studies characterize the variety Arbequina’s VOOs as having a low phenol content, no matter whether they were from different regions in Spain [14,22,23,24,25] or in other countries such as Argentina [26,27], Morocco [28], or USA [29].

Gómez-Rico et al. [30] studied the phenolic profile of olives and VOOs of many of the varieties studied in the present research: Arbequina, Cornicabra, Morisca, Picual, and Pico Limón. The lowest total phenol mean values were in VOOs of Pico Limón (198.8 mg·kg^−1^), followed by Arbequina (311.7 mg·kg^−1^), the highest values were in Cornicabra (1598.3 mg·kg^−1^) and Picual (905.7 mg·kg^−1^), and the VOOs of Morisca were intermediate in mean value (426.0 mg·kg^−1^). The Cornicabra and Picual values were greater than those of the present work, resulting from a higher secoiridoid derivative content. In the VOOs of these two varieties, those authors [30] found a predominance of the DOA and AOA forms in contrast to DLA and ALA, whereas in the remaining varieties they studied there was a predominance of the decarboxymethyl (DLA and DOA) over the aldehyde (ALA and AOA) forms, as also in our mean data presented in Table 1. For the minor phenols, the values presented in that work [30] have certain differences with those of the present study. In particular, for the variety Arbequina they found ferulic acid contents of 25.0–33.7 mg·kg^−1^, whereas in Table 1 all the varieties show mean values of less than 0.8 mg·kg^−1^.

Two of the main varieties of olives grown in Extremadura, especially because of their aptness for use in the table olive industry, are Manzanilla Cacereña and Manzanilla de Sevilla (known locally as Carrasqueña). In our study, both varieties had high phenolic contents, above 450 mg·kg^−1^. For these same varieties grown in a super-intensive framework in Elvas (Elvas, Portugal), a region on the border with Extremadura, Morales-Sillero and García [31] obtained values slightly below those of our study. In VOOs of the variety Manzanilla Cacereña, harvested by hand and with a colour index of 13.3, they obtained total phenol values of 258.6 mg·kg^−1^, while those collected by straddle harvester and with a colour index of 1.9 gave 200 mg·kg^−1^. For the variety Manzanilla de Sevilla, those authors obtained very different values between those harvested by hand and those by straddle harvester (mean total phenol values of 539.1 mg·kg^−1^, colour index 3.8 and 383.9 mg·kg^−1^, colour index 3.2, respectively). It is worth noting that Morales-Sillero and García [31] found the main phenol for VOOs of the variety Manzanilla Cacereña to be AOA at 41.4% and 37.7%, for the two cases above mentioned, whereas in our study it was DLA at 31.5%. Regarding Manzanilla de Sevilla, those workers found oleuropein derivatives (DOA and AOA) to be the majority phenols. This result coincides qualitatively with those shown in Table 1, especially in the case of the sample harvested by hand which had a greener state. In addition, for Manzanilla Cacereña, Sánchez-Casas et al. [32] obtained lower phenol contents, with values in the range 241–264 mg caffeic acid·kg^−1^.

With respect to the results presented in this section, the VOOs’ phenol compositions present characteristics that are proper of each variety, and therefore could be used as an instrument to differentiate the varietal origin of VOOs. For these reasons, they have been used in much research on the characterization of varieties [33].

### 2.2. Characterization of Fatty Acids

Regarding the FA composition (Table 2), the results show that there are clear differences between the varieties, with the varietal factor being the most decisive in determining whether or not a given FA predominates [14,34,35]. The FA values of OOs present a wide range of variability, with genetic factors explaining 73%–80% of the differences between varieties [14]. However, other factors, such as the edaphoclimatic conditions [36,37,38,39], also influence the said composition.

The main FA in all varieties was oleic, with mean values between the 63.17% of the Verdial de Badajoz VOOs and the 80.67% of Picual. The second most important (except for Morisca and Verdial de Badajoz) was palmitic, with values between the 11.62% of Picual and the 15.32% of Arbequina.

For the Morisca and Verdial de Badajoz varieties, the second main FA was linoleic, with palmitic being the third. Linoleic acid was found in the VOOs in amounts ranging from 3.08% in Picual to 17.52% in Verdial de Badajoz. Stearic was the fourth most abundant FA in the varieties studied except for the variety Arbequina in which palmitoleic was more abundant. Stearic’s mean values ranged from 1.33% in Arbequina to 2.89% in Verdial de Badajoz. Palmitoleic ranged from 0.80% in Verdial de Badajoz to 1.64% in Arbequina. Linolenic was present in amounts between 0.59% in Arbequina and 0.86% in the Morisca variety. The remaining FAs were detected in smaller quantities, although not for that less important because their concentrations will serve for the detection of spurious oils in olive oils.

To facilitate the comparison of the VOOs of different varieties, we grouped the FAs according to their degree of unsaturation, and studied the ratios between them. The greatest content of SFA and therefore the lowest of UFA was found in the Arbequina, Morisca, and Verdial de Badajoz VOOs, with mean values above 17.24%, whereas the SFA values of the varieties Manzanilla Cacereña and Picual were less than 14.52%. The greatest MUFA content was found in the VOOs of the varieties Picual (82.09%), Manzanilla Cacereña (79.94%), and Cornicabra (78.72%), while Verdial de Badajoz (64.41%), Morisca (67.32%), and Arbequina (69.45%) had the lowest values. Regarding the PUFA content, the highest values were presented by Verdial de Badajoz (18.35%), Morisca (15.40%), and Arbequina (13.26%), while the VOOs of Manzanilla Cacereña, Cornicabra, and Picual had the lowest values (≤6%).

From the values of these groups of FAs, we calculated the relations between them, finding that the UFA/SFA ratio would allow the Cornicabra, Manzanilla Cacereña, and Picual varieties to be characterized as having high values, whereas Arbequina, Morisca, and Verdial de Badajoz would have the lowest values, leaving the varieties Manzanilla de Sevilla and Pico Limón as an intermediate group. The MUFA/PUFA and SFA/PUFA ratios also allowed a similar grouping to be made of the varieties analysed.

The results we obtained for the different varieties are consistent with those published by other workers [35,40]. Those authors observed that the VOOs of Manzanilla Cacereña and Picual had the highest oleic acid contents (79.77% and 79.31%, respectively), with the mean values for Cornicabra, Manzanilla de Sevilla, Morisca, and Verdial de Badajoz being 78.34%, 75.26%, 64.30%, and 62.71%, respectively. Another aspect that coincides with the present results was that they also found greater linoleic than palmitic values for the Morisca and Verdial de Badajoz varieties.

Fuentes de Mendoz [20] studied the FA composition of the varieties Morisca and Manzanilla de Sevilla (denominated Carrasqueña in that study) grown in Extremadura. The oleic content in Morisca was found to be 64.00%, while in Manzanilla de Sevilla it was 74.70%. These values are only slightly lower than those given in Table 1 and Table 2 for the present study. The composition of the other main FAs also coincides with the present results, including the low linoleic and linolenic contents in the VOOs of Manzanilla de Sevilla but high in Morisca, and the groupings according to the level of unsaturation.

Other literature consulted shows that values similar to those of the present work had been obtained in the Catalonia and Córdoba germplasm banks [14] in which the variety Arbequina presented oleic contents of 68.20% and 65.58%, respectively. Vossen [41] found similar FA contents in this variety cultivated in Spain, Morocco, France, and Australia despite the different edaphoclimatic characteristics of each region. Interestingly, various workers studying the cultivation of Arbequina in Spain only [42,43] report greater variability in this FA than Vossen [41] found for different locations worldwide. Aranda et al. [44] found, for commercial samples of Arbequina, values of palmitic acid of 13.70%, oleic 70.60%, and linoleic 10.30%. These results are slightly different from those of our study, but similar to those reported for that variety in different zones, such as by Pardo et al. [25] in Campos de Hellín (Albacete, Spain).

Another characteristic of the variety Arbequina is that palmitic is the fourth main FA in its VOOs, with mean values above those of stearic acid. This characteristic was also observed in samples of the same variety in the Córdoba germplasm bank, although in that of Catalonia the two FAs presented equal values [14]. In addition, the palmitoleic content has been described as being greater than that of stearic in other varieties, for example, Grossal, Vimbodi, Fulla de Salze, Galega Vulgar, Piculo, and Rosciola [14].

The Cornicabra variety of the Córdoba germplasm bank [14] presents an oleic acid content of 74.30%, a value slightly lower than that shown in Table 2. This difference can be explained by the linoleic content, 7.02%, which is somewhat greater than in the present study. As do Ramos-Escudero et al. [18], other researchers too have obtained mean oleic values for this variety greater than those found in our work. Thus, Salvador et al. [45] obtained a mean oleic value of 80.84%, although the linoleic content of 4.66% was similar to that obtained in the present study. The main difference with the data obtained by Salvador et al. [45,46] was in the palmitic acid values −9.20% in their case, well below the mean of 12.12% obtained in the present study. Aranda et al. [44] and Pardo et al. [25] also found values similar to those of Salvador et al. [45,46] in different locations of Castilla la Mancha. This difference in palmitic acid content in Cornicabra VOOs from Castilla la Mancha had previously been observed in other studies conducted in Extremadura [40].

This same germplasm bank gives values of oleic acid for the Manzanilla Cacereña variety (75.33%) that are lower than those obtained in our study, and a linoleic acid content that is greater. Other results, such as those cited above of Sánchez-Casas et al. [40] and Sánchez-Casas [35] in Manzanilla Cacereña olive groves in northern Cáceres, and of Morales-Sillero and García [31] in super-intensive cultivation in the Portuguese locality of Elvas, are identical, thus confirming the values obtained in this present study.

For the variety Manzanilla de Sevilla, the two germplasm banks give oleic values of 71.97% and 69.85%, respectively, slightly lower than the data obtained in the present study. These differences could be due to the palmitic and linoleic contents which are both higher than those we obtained. However, Pardo et al. [25] in Campos de Hellin (Albacete, Spain) and Morales-Sillero and García [31] in Elvas (Elvas, Portugal), the latter in a super-intensive framework, obtained values similar to those given in Table 1 for all the main FAs.

The Pico Limón variety was described by Tous et al. [14] as having a similar oleic content to that of Manzanilla de Sevilla, as was also observed in this present study. In the Córdoba germplasm bank, slightly lower mean oleic acid values were obtained (69.85%) because of a greater linoleic acid content.

The variety Picual presented very high oleic contents in both the Catalonia and Córdoba germplasm banks, with values of 78.28% and 78.34%, respectively, slightly lower than those obtained in our study. Aranda et al. [44], from commercial samples of this variety, found values similar to those of the two germplasm banks, with the content of palmitic at 10.60%, oleic at 78.90%, and linoleic at 4.53%, results which differ only slightly from those presented in the present work.

Finally, the Verdial de Badajoz variety is identified in the Córdoba germplasm bank as a variety with very low oleic content [14], having mean values of 57.44%. Whereas such low values were observed in some specific samples analysed in the present study, the mean values observed for olives grown in Extremadura were higher.

Other published studies have reported values similar to those found in this present study. Thus, Ramos-Escudero et al. [18], for samples bought retail, obtained values for Manzanilla Cacereña of 79.20% for oleic, 10.10% for palmitic, 5.50% for linoleic, and 2.80% for stearic acid. The retail Cornicabra variety oils gave values of oleic 82.50%, palmitic 8.90%, linoleic 3.30%, and stearic 3.20%, and Arbequina gave oleic 70.10%, palmitic 14.50%, linoleic 10.30%, and stearic 2.00%. These last two monovarietal samples had an oleic acid content slightly greater than those that we obtained in this present study, even greater than the values obtained by Tous et al. [14] from the two germplasm banks in Spain, while the retail sample of Manzanilla Cacereña presented values similar to those in Table 1, coinciding also with the results reported by other workers [20,35,40].

Aguilera et al. [47] state that the main differences between cultivars located in different regions are primarily in the palmitic acid contents, whereas Ouni et al. [48] found that, for the Chétoui variety, the altitude at which the trees were growing most clearly affected the oleic and linoleic acids, with the MUFA/PUFA ratio declining the closer the trees are to sea level. These differences would explain, for example, the different FA composition of varieties grown in regions far apart.

### 2.3. Oxidative Stability

Table 3 presents the OS results. The greatest values of the induction time, and therefore the greatest OS, were found in the Picual and Cornicabra variety oils, with mean values above 58 h, significantly greater (*p* < 0.05) than the other varieties.

The VOOs of the varieties Manzanilla de Sevilla and Manzanilla Cacereña also showed high values of OS, with mean values greater than 50 h. The variety Pico Limón gave intermediate OS values, reaching a mean value of about 40 h. The VOOs of the other varieties presented low OS values, less than 35 h: Morisca with mean values of 31.5 h, and Arbequina and Verdial de Badajoz below 30 h.

These results are lower than those obtained by Tous et al. [14] and Uceda et al. [49], although qualitative coincidence is observed in the order of the varieties depending on the magnitude of this parameter. Tous et al. [14] found that the VOOs of Picual and Cornicabra had the greatest oxidative resistance of the 74 varieties of the CIFA’s “Alameda del Obispo” World Olive Germplasm Bank in Córdoba, reaching 140.6 and 118.7 h, respectively. For Manzanilla Cacereña they calculated 77.1 h, for Manzanilla de Sevilla 57.5 h, for Pico Limón 44.4 h, for Arbequina 38.3 h, and finally for Verdial de Badajoz 23.3 h. They did not provide any data for Morisca. Qualitatively, the present study’s data are consistent with those results.

The OS of Arbequina oils has been much studied. The values obtained have been low: Tous et al. [14] and Uceda et al. [49] found it to be less than 45 h, while Yousfi et al. [24] found values of 29.8 h when the fruit had been harvested by hand and 20.6 h when a straddle harvester had been used.

Pardo et al. [25] studied different varieties grown in Albacete (Spain). They found greater OS in samples obtained using Oliomio equipment than in the industrial samples. For the variety Arbequina, they report values of 37–48 h when the olives had been processed with Oliomio versus 32–39 h obtained for samples from olive mills. Similarly, the Picual VOOs produced with Oliomio gave values of 67–86 versus 71–75 h for mill samples. Other results obtained by Pardo et al. [25] are consistent with those we obtained in this present study since they obtained values of 72–86 h for the variety Cornicabra and 44–58 h for Manzanilla de Sevilla. Gómez-Rico et al. [50] also observed a great difference in OS between Cornicabra (≈30 h) and Morisca (<6 h).

Morales-Sillero and García [31] found the OS values for Manzanilla Cacereña and Manzanilla de Sevilla in olive groves in a super-intensive framework in Elvas (Elvas, Portugal) to depend on the harvesting system. The variety Manzanilla Cacereña offers a mean value of 98.6 h with harvesting by hand and colour index of 3.8, and of 85.2 h with harvesting by hand and colour index of 3.2. The variety Manzanilla de Sevilla presented values than Manzanilla Cacereña: mean values of 113.5 h with harvesting by hand and colour index of 13.3, and of 89.0 h with harvesting by hand and colour index of 1.9. These results are in line with those obtained for Manzanilla de Sevilla in a green state by Luaces et al. [51], surpassing in OS the variety Picual with 127.7 h versus 114.0 h.

The variety Verdial de Badajoz has been described as one of those giving lower OS values [52]. Tous et al. [14] obtained values of less than 25 h in oils obtained from the olive trees located in the Córdoba germplasm bank.

### 2.4. Relation between Phenolic Compounds, Fatty Acids, and Oxidative Stability

We studied the relationship of the different FA and phenolic compounds with OS (Table 4). For the variety Arbequina, we obtained high values of the Pearson coefficient for the relationship of OS with the DOA content (*R* = 0.768, *p* = 0.001), ferulic acid (*R* = −0.624, *p* = 0.013), total phenols (*R* = 0.634, *p* = 0.011), secoiridoid derivatives (*R* = 0.672, *p* = 0.006), and *o*-diphenols (*R* = 0.706, *p* = 0.003). The main phenol in the VOOs of Arbequina, DOA (Table 1), accounts for nearly 40% of the phenols, and is a secoiridoid derivative and an *o*-diphenol, so that the high value of the Pearson coefficient between these groups of phenols and OS is evident.

For the VOOs of the variety Cornicabra, we found a high Pearson coefficient for the relationship between OS and the luteolin content (*R* = −0.623, *p* = 0.010). For other compounds studied, such as the content in linoleic acid and some phenolic compounds, the Pearson coefficients were not high, although they were significant (*p* < 0.05). The VOOs of Manzanilla Cacereña showed few significant relationships, outstanding being that with the linoleic content (*R* = −0.441, *p* = 0.031).

Manzanilla de Sevilla showed high Pearson coefficients for the relationships with the oleic content (*R* = 0.631, *p* = 0.009) and some phenolic compounds such as *p*-coumaric acid (*R* = −0.691, *p* = 0.003), ferulic acid (*R* = −0.667, *p* = 0.005), luteolin (*R* = −0.816, *p* < 0.001), and apigenin (*R* = −0.823, *p* < 0.001).

The Morisca VOOs presented high values of Pearson coefficients with the DOA content (*R* = 0.685, *p* = 0.002) and *o*-diphenols (*R* = 0.636, *p* = 0.006), with DOA being the main phenol (Table 1), representing 47% of the total phenols.

Verdial de Badajoz had the VOOs with the lowest OS values of all the varieties studied. This was significantly correlated with the content in palmitic (*R* = −0.607, *p* = 0.013), palmitoleic (*R* = −0.648, *p* = 0.007), stearic (*R* = 0.729, *p* = 0.001), oleic (*R* = 0.884, *p* < 0.001), linoleic (*R* = −0.806, *p* < 0.001), hydroxytyrosol (*R* = 0.605, *p* = 0.013), DLA (*R* = 0.798, *p* < 0.001), total phenols (*R* = 0.670. *p* = 0.004), and secoiridoid derivatives (*R* = 0.669, *p* = 0.004). The Pico Limón and Picual VOOs showed no significant relationships between the OS and the different phenolic compound or FA contents.

Other research studies have found the FA’s to have a major influence on OS, as was also observed in the present results for Arbequina. Thus, Gracia et al. [22] found a Pearson coefficient between total phenols and OS of *R* = 0.78. Salvador et al. [46] related different variables of the VOOs of the variety Cornicabra with OS. For the relationship with the total phenols they found a very high Pearson coefficient: *R* = 0.8374. Gutiérrez-Rosales et al. [8] studied the relationship between OS and the different chemical components of the VOOs of Picual and Hojiblanca. Regarding Picual, they obtained high Pearson coefficients with the content in total phenols (*R* = 0.983, *p* < 0.01), *o*-diphenols (*R* = 0.991, *p* < 0.01), palmitic acid (*R* = 0.973, *p* < 0.05), linoleic acid (*R* = 0.979, *p* < 0.01), and linoleic acid (*R* = 0.979, *p* < 0.01). In addition, for Picual VOOs, Aparicio et al. [7] obtained important relationships with total phenols (*R* = 0.87), *o*-diphenols (*R* = 0.77), and the oleic/linoleic acid ratio (*R* = 0.71).

**Figure 1:**
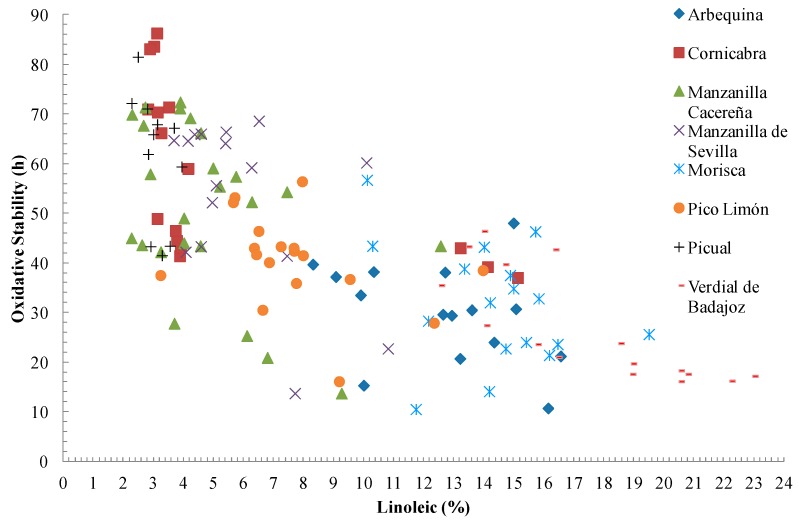
Relationship between oxidative stability (h) and the linoleic acid content (%) for the VOOs analysed.

In considering all the samples obtained without discriminating by variety (*n* = 136), we found the OS to be clearly correlated with the oleic (*R* = 0.688, *p* < 0.001) and linoleic (*R* = −0.710. *p* < 0.001) acid contents (Figure 1). The wide range of oleic, and therefore of linoleic, acid content allowed the varieties to be differentiated, and, in general, is the factor that best explains the differences in the OS of the VOOs. There are various studies in the literature that are in line with these results—the study for example of Rotondi et al. [9] of the variety Nostrana di Brisighella at four stages of ripeness. In addition, Gracia et al. [22] studied Arbequina growing in Montañana (Zaragoza, Spain), finding similar OS values for Arbequina and Empeltre OOs despite the greater total phenol content of the latter. They explained this behaviour as being due to the PUFA content, although they did not exclude the influence of the high peroxide value presented by the oils.

In this section, it has been seen in general that the OS of the VOOs of Arbequina, Morisca, and Verdial de Badajoz (the three with low oleic acid values) are influenced by the phenol content, especially by the main phenol which usually reaches about 50% of the total phenols. However, the varieties with greater oleic and lesser linoleic contents presented little influence of phenolic compounds on OS, while not ruling out the possibility of more complex relationships between FAs and phenolics.

**Figure 2:**
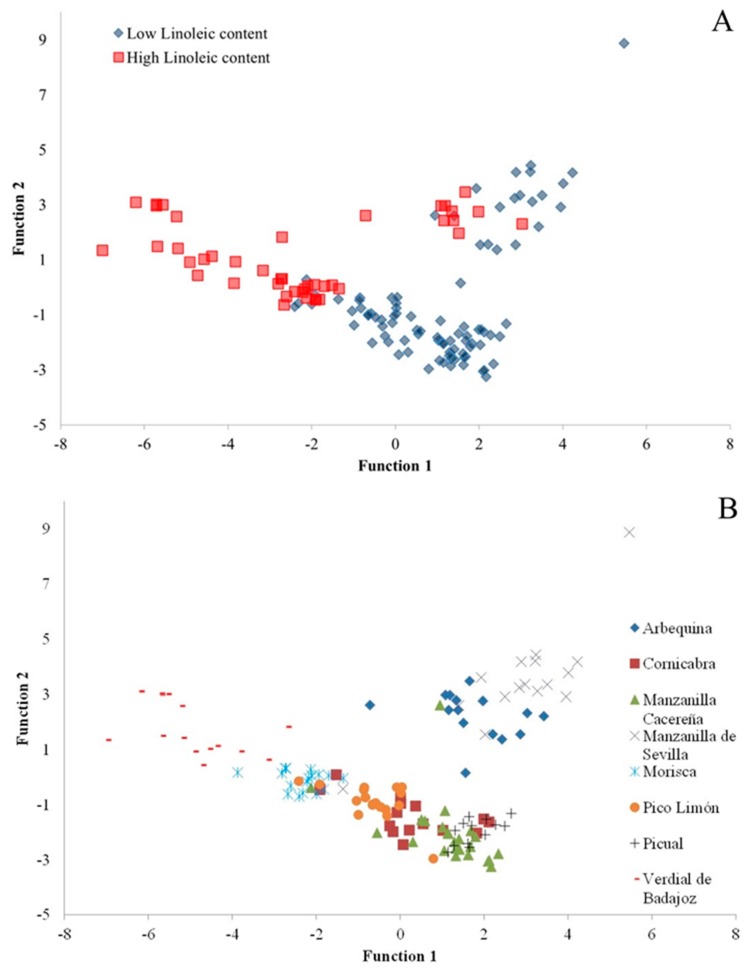
Scatter plot on axes representing the first two discriminant functions showing (**A**) the samples with high (≥12.5%) and low (<12.5%) linoleic acid contents and (**B**) the different cultivars. Function coefficients are shown in Table 5.

As one observes in Figure 2A, this is supported by the fact that if only the samples with linoleic contents greater than or equal to 12.5% are considered then maximal Pearson coefficients for the relationships between the phenolics and OS are obtained. This value of ≥12.5% linoleic acid content would thus seem to be a good criterion with which to discriminate between samples. Of the total of VOO samples analysed in this study, there are 47 that satisfy this criterion: 10× Arbequina, 3× Cornicabra, 1× Manzanilla Cacereña, 13× Morisca, 1× Pico Limón, and 16× Verdial de Badajoz. Then, in relating the OS with the phenolic compound contents, one obtains high Pearson coefficients: with total phenols (*R* = 0.662, *p* < 0.001), with secoiridoid derivatives (*R* = 0.669, *p* < 0.001), and with *o*-diphenols (*R* = 0.674, *p* < 0.001), slightly greater than the Pearson coefficients obtained between the OS and the oleic (*R* = 0.656, *p* < 0.001) and linoleic (*R* = −0.598, *p* < 0.001) acid contents. In considering other ranges of linoleic acid content, such as “≤5.0%” or “5.0% < linoleic < 12.5%”, we obtained no significant Pearson coefficients for the relationships with phenolic compounds, while the Pearson coefficients for the relationships between OS and oleic and linoleic acids were greater and more significant.

As mentioned above, it is possible that there exist more complex relationships such as, for example, those proposed by Salvador et al. [45] who obtained a high Pearson coefficient for samples of Cornicabra during three successive seasons (*n* = 74) in relating stability with the total phenol + α-tocopherol + FA content (more specifically with the linoleic + linolenic versus oleic relationship). In the same vein, Aparicio et al. [7] found for Picual and Hojiblanca a regression model (*R* = 0.83, *n* = 79) in which the OS is related to the same parameters except that the influence of the FAs was determined by the oleic/linoleic ratio. Mateos et al. [10] reached the same conclusions, and indicated that the position and conformation of the FAs in the triacylglyceride is even more important than the simple composition of the FA profile.

### 2.5. Discriminant Analysis

We subjected the data of the 134 samples analysed to a linear discriminant analysis (LDA) in order to obtain a model classifying the VOOs that we had studied. Table 5 lists the standardized coefficients of the seven canonical discriminant functions obtained. These include the fatty acids palmitoleic, margaric, linoleic, and eicosenoic, and the phenols hydroxytyrosol, tyrosol, *p*-coumaric acid, hydroxytyrosol acetate, DOA, DLA, and the sum of lignans + cinnamic acid. In the first function, there stand out because of their importance linoleic acid and the phenols DOA and DLA, and in the second, the margaric acid and tyrosol contents. Figure 2B plots the VOOs of the different varieties in terms of these two functions of the proposed model. This model, as shown in Table 6, correctly classifies 96.3% of the cases of different varieties, which indicates the suitability of the proposed discriminant analysis. An exception is the Cornicabra variety, 87.5% of whose data are correctly classified. However, there is 100% correct classification of the VOOs of the varieties Arbequina, Manzanilla de Sevilla, Morisca, Picual, and Verdial de Badajoz.

## 3. Material and Methods

### 3.1. Samples

Samples of the main varieties of olives grown in Extremadura (Spain)—Manzanilla Cacereña, Manzanilla de Sevilla, Cornicabra, Pico Limón, Morisca, Verdial de Badajoz, and Arbequina—were collected on three different harvesting dates from at least three different groves in different locations, using one or two olive trees for each harvesting date.

### 3.2. Extraction of Virgin Olive Oils

The olives were harvested by hand and milled using the Abencor^®^ system (MC2 Ingenierias y Sistemas, Sevilla, Spain), following the procedure described by Martinez et al. [53]. This system comprises three essential elements: mill, thermobeater, and pulp centrifuge. The extraction was carried out at 28 °C with kneading for 30 min without headspace control. After milling, the oil was decanted off, filtered, transferred to a glass bottle, and stored at −30 °C.

### 3.3. Determination of Phenolic Compounds

Phenols were assayed following the method described by Mateos [4] and Mateos et al. [54]. All reagents were HPLC-grade from Panreac (Barcelona, Spain). Distilled water was de-ionized in a Milli-Q system (Millipore, Bedford, MA, USA). Phenols were extracted through an SPE Diol column (Sep-Pak^®^ Vac 3cc, Waters) with 6 mL hexane and 2.5 mL hexane:ethyl acetate (9:1) per 2.5 g of oil, using 10 mL of methanol as eluent. The resulting extract was vacuum evaporated, and the residue dissolved in 0.5 mL methanol:water (1:1). The clear solution was kept at room temperature for 4 h before HPLC assay.

The HPLC system consisted of an Agilent 1200 Series isocratic and binary gradient pump, Agilent 1200 Series autosampler, Agilent 1200 Series thermostatted column compartment, and Agilent 1200 Series diode array and multiple wavelength detector managed by Agilent ChemStation. The analytical column was an Eclipse XDB-C18 (Agilent Technologies, Santa Clara, CA, USA), particle size 5 µm, length 150 mm, and internal diameter 4.6 mm. The HPLC assay parameters were: injection volume 5 µL, column temperature 30 °C, flow rate 1 mL/min. The total run time was 55 min. Two eluents were used: orthophosphoric acid:water (99.5:0.5) and methanol:acetonitrile (50:50) with gradients 95:5 at *t* = 0 min, 30:70 at *t* = 25 min, 38:62 at *t* = 40 min, 45:55 at *t* = 45 min, 52:48 at *t* = 50 min, and 100:0 at 55 min.

Phenols were assayed at 280 nm using syringic acid and at 335 nm using *o*-coumaric acid as internal standards, both of which were purchased from Sigma-Aldrich (St. Louis, MO, USA). The response factors were determined following Mateos et al. [55]. The results are expressed in mg oil kg h^−1^.

### 3.4. Fatty Acid Methylation and Analyses

Methyl esters were prepared according to the standard EU official method [56]. The relative composition of the VOO samples was determined as percentages of total fatty acids using a Hewlett Packard gas chromatograph equipped with a flame ionization detector (FID). A polyethylene glycol fused silica capillary column, CarboWax (60 m × 0.25 mm × 0.25 µm film thickness) (Agilent Technologies, Santa Clara, CA, USA), was used. The carrier gas was helium (99.999%, Air Liquid), at a column flow of 20 mL·min^−1^. The temperature of the detector was 250 °C, and the column was maintained at 150 °C for 10 min, and then heated at a rate of 4 °C·min^−1^ to 230 °C, at which it was maintained for 10 min.

### 3.5. Determination of Oxidative Stability

OS was evaluated using a Rancimat 743 apparatus (Metrohm Co., Basel, Switzerland), measuring the oxidative-induction time. A flow of air (15 L·h^−1^) was bubbled through 2.5 g of oil heated to 100 °C. The Rancimat stability was taken to be the time needed for an abrupt change in conductivity of an aqueous solution in which the volatile compounds resulting from oxidation of the oil were collected [57].

### 3.6. Statistical Analysis

All statistical analyses were performed using the IBM^®^ SPSS^®^ vn 21.0 Statistics software package (SPSS Inc., Chicago, IL, USA). Mean values obtained for the variables studied in the different groups were compared by a one-way ANOVA (Duncan’s multiple range test), taking differences to be significant at the level of *p* < 0.05. A simple linear correlation analysis was applied to examine possible relationships between variables. A stepwise linear discriminant analysis (LDA) was used to classify the cultivar samples into homogeneous groups.

## 4. Conclusions

We have analysed and characterized the phenol composition and the FA content of VOOs of the principal varieties of olives grown in Extremadura. The said content allows the varieties to be characterized and classified into different groups: (1) varieties with high OS, rich in oleic acid and a medium-high phenol content (Cornicabra, Picual, and Manzanilla Cacereña); (2) varieties with medium oleic acid and medium-high phenol contents (Manzanilla de Sevilla and Pico Limón); and (3) varieties with low OS, either because of a low phenol content (Arbequina) or because of a high linoleic acid content despite their VOOs having a high phenol content.

It has been shown that the most influential factor for the oils’ OS, excluding the variety factor, is the linoleic acid content, although the concentration of phenolic compounds is as influential as the linoleic and oleic content for VOOs with a high linoleic content (≥12.5%).

The results we obtained are of great interest for the VOO producing and packaging sector because they will help in decision-making about the commercial destinations of certain VOOs depending on their potential oxidative resistance, and therefore shelf-life, as well as their nutritional quality.

## Figures and Tables

**Table 1:** Mean values of phenolic concentrations (mg·kg^−1^) in the VOOs of the Arbequina, Cornicabra, Manzanilla Cacereña, Manzanilla de Sevilla, Morisca, Pico Limón, Picual, and Verdial de Badajoz varieties. Different lower case letters in the same row indicate a significant difference at the *p* < 0.05 level according to the Duncan test, as well as the belonging to a different homogeneity group. The absence of a letter indicates that the ANOVA found no significant effect among the different varieties (data expressed as mean ± standard deviation).

Phenols and Phenol Groups (mg·kg^−1^)	Arbequina	Cornicabra	Manzanilla Cacereña	Manzanilla de Sevilla	Morisca	Pico Limón	Picual	Verdial de Badajoz
Hydroxytyrosol	1.6 ± 1.8 ^a^	10.2 ± 12.7 ^b^	8.5 ± 9.3 ^a,b^	6.2 ± 3.9 ^a,b^	6.7 ± 3.8 ^a,b^	12.4 ± 16.5 ^b^	12.8 ± 9.6 ^b^	8.0 ± 10.8 ^a,b^
Tyrosol	1.3 ± 1.0 ^a^	9.3 ± 12.5 ^b,c^	13.0 ± 8.5 ^c^	7.1 ± 2.8 ^a,b,c^	4.9 ± 2.0 ^a,b^	9.7 ± 13.7 ^b,c^	7.8 ± 4.4 ^b,c^	10.0 ± 10.2 ^b,c^
Vanillic acid	1.2 ± 0.9 ^b,c^	0.2 ± 0.3 ^a^	0.5 ± 0.3 ^a^	1.2 ± 1.4 ^b,c^	0.5 ± 0.4 ^a^	0.7 ± 0.6 ^a,b^	1.3 ± 0.7 ^c^	0.4 ± 0.4 ^a^
Vanillin	0.0 ± 0.0	0.0 ± 0.0	0.0 ± 0.0	0.0 ± 0.0	0.0 ± 0.0	0.0 ± 0.1	0.0 ± 0.1	0.0 ± 0.0
*p*-Coumaric acid	0.3 ± 0.3 ^a^	0.3 ± 0.4 ^a^	0.7 ± 0.4 ^a,b^	1.5 ± 0.7 ^c^	2.2 ± 1.3 ^d^	0.3 ± 0.5 ^a^	0.7 ± 0.3 ^a,b^	1.0 ± 0.7 ^b^
Hydroxytyrosol acetate	12.0 ± 11.6 ^b^	3.1 ± 6.8 ^a^	1.7 ± 2.1 ^a^	5.1 ± 2.9 ^a^	13.4 ± 11.0 ^b^	0.4 ± 1.2 ^a^	3.5 ± 2.8 ^a^	4.3 ± 11.1 ^a^
DOA	77.4 ± 84.3 ^a^	230.4 ± 138.6 ^c,d^	117.3 ± 123.0 ^a,b^	151.6 ± 93.4 ^a,b,c^	259.3 ± 184.1 ^d^	135.3 ± 123.3 ^a,b,c^	95.4 ± 54.9 ^a,b^	187.7 ± 213.7 ^b,c,d^
Tyrosol acetate	1.0 ± 1.9	1.2 ± 2.6	0.2 ± 1.0	0.6 ± 1.5	0.8 ± 2.4	0.2 ± 0.7	0.5 ± 1.9	0.0 ± 0.0
DLA	41.8 ± 36.9 ^a^	217.5 ± 139.2 ^c,d^	147.9 ± 135.4 ^b,c^	96.0 ± 70.3 ^a,b^	191.2 ± 126.7 ^c,d^	98.0 ± 80.5 ^a,b^	69.1 ± 41.5 ^a,b^	246.9 ± 214.7 ^d^
Lignans + cinnamic acid	28.8 ± 14.6 ^b^	10.6 ± 9.8 ^a^	6.2 ± 6.5 ^a^	11.1 ± 4.8 ^a^	10.2 ± 5.2 ^a^	36.5 ± 45.0 ^b^	7.0 ± 8.4 ^a^	33.1 ± 16.5 ^b^
AOA	25.0 ± 5.2 ^a^	94.6 ± 107.9 ^b,c,d^	89-2 ± 71.4 ^a,b,c,d^	150.7 ± 94.2 ^d^	47.4 ± 37.8 ^a,b^	77.7 ± 128.2 ^a,b,c^	127.1 ± 120.2 ^c,d^	39.4 ± 39.8 ^a,b^
ALA	6.5 ± 16.6 ^a^	53.8 ± 75.0 ^c,d,e^	79.9 ± 73.8 ^e^	61.4 ± 65.0 ^d,e^	10.6 ± 18.0 ^a,b^	30.8 ± 41.5 ^a,b,c,d^	49.4 ± 61.0 ^b,c,d,e^	14.7 ± 25.2 ^a,b,c^
Ferulic acid	0.2 ± 0.3 ^a^	0.0 ± 0.0 ^a^	0.0 ± 0.0 ^a^	0.8 ± 1.2 ^b^	0.2 ± 0.2 ^a^	0.2 ± 0.5 ^a^	0.1 ± 0.2 ^a^	0.4 ± 0.7 ^a^
Luteolin	2.5 ± 5.5	1.0 ± 1.4	2.5 ± 3.7	2.7 ± 4.0	2.2 ± 2.8	3.4 ± 5.4	4.7 ± 7.9	3.5 ± 4.3
Apigenin	0.7 ± 1.4	0.4 ± 0.7	1.1 ± 1.7	1.4 ± 2.0	0.7 ± 1.0	1.2 ± 1.9	1.1 ± 1.3	1.1 ± 1.6
Total phenols	200.2 ± 177.1 ^a^	632.6 ± 405.9 ^b^	468.8 ± 348.2 ^b^	497.5 ± 284.1 ^b^	550.2 ± 346.8 ^b^	406.8 ± 415.2 ^a,b^	380.4 ± 252.8 ^a,b^	551.4 ± 497.1 ^b^
Secoiridoid derivatives	150.7 ± 167.4 ^a^	596.3 ± 375.6 ^b^	434.3 ± 340.9 ^b^	459.8 ± 277.3 ^b^	508.5 ± 341.5 ^b^	341.8 ± 346.1 ^a,b^	340.9 ± 246.2 ^a,b^	488.7 ± 467.4 ^b^
*o*-Diphenols	118.5 ± 137.6 ^a^	339.3 ± 217.5 ^b^	219.3 ± 176.3 ^a,b^	316.4 ± 168.6 ^b^	329.0 ± 213.4 ^b^	229.1 ± 253.7 ^a,b^	243.4 ± 162.7 ^a,b^	243.8 ± 263.3 ^a,b^

**Table 2:** The FA methyl ester composition (%) of VOOs of the Arbequina, Cornicabra, Manzanilla Cacereña, Manzanilla de Sevilla, Morisca, Pico Limón, Picual, and Verdial de Badajoz varieties. Different lower case letters in the same row indicate a significant difference at the *p* < 0.05 level according to the Duncan test, as well as the belonging to a different homogeneity group. The absence of a letter indicates that the ANOVA found no significant effect among the different varieties (data expressed as mean ±standard deviation).

Parameter	Arbequina	Cornicabra	Manzanilla Cacereña	Manzanilla de Sevilla	Morisca	Pico Limón	Picual	Verdial de Badajoz
Myristic	0.02 ± 0.01	0.01 ± 0.01	0.02 ± 0.02	0.01 ± 0.01	0.02 ± 0.01	0.01 ± 0.01	0.01 ± 0.01	0.01 ± 0.01
Myristoleic	0.01 ± 0.01	0.00 ± 0.01	0.00 ± 0.01	0.00 ± 0.01	0.00 ± 0.01	0.00 ± 0.00	0.00 ± 0.01	0.00 ± 0.00
Palmitic	15.32 ± 1.41 ^d^	12.12 ± 2.06 ^a^	12.27 ± 1.20 ^a,b^	13.22 ± 1.39 ^b,c^	13.88 ± 0.89 ^c^	13.22 ± 1.26 ^b,c^	11.62 ± 1.18 ^a^	13.51 ± 1.68 ^c^
Palmitoleic	1.64 ± 0.24 ^d^	1.00 ± 0.32 ^b^	1.12 ± 0.22 ^b,c^	1.28 ± 0.23 ^c^	1.06 ± 0.18 ^b^	1.17 ± 0.21 ^b,c^	1.02 ± 0.17 ^b^	0.80 ± 0.28 ^a^
Margaric	0.11 ± 0.01 ^c^	0.05 ± 0.01 ^a,b^	0.04 ± 0.02 ^a^	0.17 ± 0.06 ^d^	0.05 ± 0.01 ^a,b^	0.04 ± 0.01 ^a^	0.04 ± 0.01 ^a^	0.06 ± 0.01 ^b^
Margaroleic	0.23 ± 0.02 ^b^	0.08 ± 0.01 ^a^	0.08 ± 0.04 ^a^	0.28 ± 0.07 ^c^	0.07 ± 0.01 ^a^	0.11 ± 0.12 ^a^	0.09 ± 0.01 ^a^	0.07 ± 0.01 ^a^
Stearic	1.33 ± 0.48 ^a^	2.44 ± 1.10 ^c,d^	1.66 ± 0.73 ^a,b^	2.79 ± 0.98 ^d^	2.70 ± 0.83 ^d^	2.07 ± 0.64 ^b,c^	1.96 ± 0.88 ^b,c^	2.89 ± 1.03 ^d^
Oleic	67.25 ± 4.31 ^b^	77.36 ± 6.07 ^d^	78.40 ± 3.37 ^d,e^	74.65 ± 2.730 ^c^	65.95 ± 3.16 ^b^	74.14 ± 3.19 ^c^	80.67 ± 1.95 ^e^	63.17 ± 3.59 ^a^
Linoleic	12.66 ± 2.60 ^d^	5.32 ± 4.25 ^b^	4.84 ± 2.41 ^a,b^	5.95 ± 2.11 ^b,c^	14.54 ± 2.42 ^e^	7.71 ± 2.45 ^c^	3.08 ± 0.46 ^a^	17.52 ± 3.32 ^f^
Linolenic	0.59 ± 0.13 ^a^	0.67 ± 0.25 ^a^	0.67 ± 0.12 ^a^	0.64 ± 0.22 ^a^	0.86 ± 0.09 ^b^	0.65 ± 0.08 ^a^	0.65 ± 0.12 ^a^	0.83 ± 0.17 ^b^
Arachidic	0.34 ± 0.07 ^a^	0.45 ± 0.10 ^b,c^	0.37 ± 0.05 ^a^	0.48 ± 0.05 ^c^	0.45 ± 0.04 ^b,c^	0.43 ± 0.04 ^b^	0.36 ± 0.02 ^a^	0.55 ± 0.04 ^d^
Eicosenoic	0.31 ± 0.07 ^b^	0.27 ± 0.03 ^a^	0.34 ± 0.04 ^b^	0.33 ± 0.12 ^b^	0.24 ± 0.02 ^a^	0.25 ± 0.05 ^a^	0.31 ± 0.05 ^b^	0.37 ± 0.08 ^c^
Behenic	0.13 ± 0.03 ^b^	0.14 ± 0.01 ^b^	0.13 ± 0.03 ^b^	0.14 ± 0.03 ^b^	0.13 ± 0.02 ^a,b^	0.13 ± 0.03 ^a,b^	0.11 ± 0.01 ^a^	0.14 ± 0.02 ^b^
Lignoceric	0.05 ± 0.02 ^a^	0.07 ± 0.02 ^b^	0.07 ± 0.03 ^b^	0.07 ± 0.02 ^b^	0.05 ± 0.02 ^a^	0.07 ± 0.02 ^b^	0.07 ± 0.02 ^b^	0.08 ± 0.02 ^b^
SFA	17.30 ± 1.80 ^d^	15.29 ± 2.47 ^a,b^	14.56 ± 1.59 ^a^	16.88 ± 1.34 ^c,d^	17.27 ± 1.43 ^d^	15.97 ± 1.65 ^b,c^	14.18 ± 1.56 ^a^	17.24 ± 1.29 ^d^
UFA	82.70 ± 1.80 ^a^	84.71 ± 2.47 ^c,d^	85.44 ± 1.59 ^d^	83.13 ± 1.34 ^a,b^	82.73 ± 1.43 ^a^	84.03 ± 1.65 ^b,c^	85.82 ± 1.56 ^d^	82.76 ± 1.29 ^a^
MUFA	69.45 ± 4.12 ^b^	78.72 ± 6.07 ^d,e^	79.94 ± 3.33 ^e,f^	76.54 ± 2.74 ^c,d^	67.32 ± 3.12 ^b^	75.68 ± 3.10 ^c^	82.09 ± 1.92 ^f^	64.41 ± 3.36 ^a^
PUFA	13.26 ± 2.67 ^d^	5.99 ± 4.35 ^b^	5.50 ± 2.42 ^a,b^	6.59 ± 2.19 ^b,c^	15.40 ± 2.45 ^e^	8.35 ± 2.46 ^c^	3.73 ± 0.54 ^a^	18.35 ± 3.31 ^f^
UFA/SFA	4.84 ± 0.64 ^a^	5.72 ± 1.22 ^b,c^	5.95 ± 0.78 ^c^	4.96 ± 0.49 ^a^	4.83 ± 0.51 ^a^	5.33 ± 0.74 ^a,b^	6.14 ± 0.82 ^c^	4.83 ± 0.43 ^a^
MUFA/PUFA	5.52 ± 1.55 ^a^	17.59 ± 6.86 ^c^	16.89 ± 6.26 ^c^	12.68 ± 3.53 ^b^	4.52 ± 1.00 ^a^	9.93 ± 3.67 ^b^	22.49 ± 3.64 ^d^	3.65 ± 0.86 ^a^
SFA/PUFA	3.67 ± 0.81 ^d^	2.85 ± 0.94 ^a,b^	2.52 ± 0.59 ^a^	3.46 ± 0.60 ^c,d^	3.64 ± 0.65 ^d^	3.08 ± 0.66 ^b,c^	2.38 ± 0.55 ^a^	3.62 ± 0.61 ^d^

**Table 3:** The oxidative stability (Rancimat, h) of the VOOs of the varieties studied. Different letters indicate a significant difference. The Duncan test with a 95% confidence level (*p* < 0.05) was used to discriminate between means.

Varieties	Mean ± Standard Deviation
Arbequina	29.8 ± 10.0 ^a,b^
Cornicabra	58.4 ± 17.4 ^c,d^
Manzanilla Cacereña	51.8 ± 17.0 ^c^
Manzanilla de Sevilla	53.2 ± 16.4 ^c^
Morisca	31.5 ± 12.1 ^a,b^
Pico Limón	40.3 ± 9.4 ^b^
Picual	66.6 ± 20.8 ^d^

**Table 4:** Regression coefficients between oxidative stability and phenolics (mg·kg^−1^) of the oils of all the varieties studied. In boldface are the coefficients above 0.6 in absolute value.

Parameter	All Samples (*n* = 136)	Arbequina (*n* = 15)	Cornicabra (*n* = 16)	Manzanilla Cacereña (*n* = 25)	Manzanilla de Sevilla (*n* = 16)	Morisca (*n* = 17)	Pico Limón (*n* = 18)	Picual (*n* = 13)	Verdial de Badajoz (*n* = 16)
Palmitic	−0.381 ^1^	−0.274	0.049	0.050	−0.109	−0.052	0.405	−0.133	**−0.607**
	<0.001 ^2^	0.322	0.857	0.815	0.688	0.844	0.095	0.666	0.013
Palmitoleic	−0.124	−0.438	0.366	−0.267	0.071	−0.088	−0.557	−0.103	**−0.648**
	0.153	0.102	0.164	0.207	0.793	0.738	0.016	0.739	0.007
Stearic	−0.147	−0.482	−0.470	0.058	−0.490	−0.060	0.193	−0.570	**0.729**
	0.089	0.069	0.066	0.789	0.054	0.820	0.444	0.042	0.001
Oleic	**0.688**	0.359	0.470	0.278	**0.631**	0.286	0.102	0.407	**0.884**
	<0.001	0.189	0.066	0.189	0.009	0.266	0.688	0.167	<0.001
Linoleic	**−0.710**	−0.357	−0.587	−0.441	−0.499	−0.334	−0.378	−0.322	**−0.806**
	<0.001	0.191	0.017	0.031	0.049	0.190	0.122	0.284	<0.001
Hydroxytyrosol	0.094	0.413	−0.276	−0.009	0.038	0.459	0.125	−0.523	**0.605**
	0.278	0.126	0.301	0.967	0.888	0.064	0.620	0.067	0.013
Tyrosol	0.086	0.213	−0.245	−0.002	−0.105	0.259	0.066	−0.586	0.544
	0.319	0.445	0.360	0.994	0.697	0.315	0.794	0.035	0.029
*p*-Coumaric ac.	−0.238	−0.429	−0.572	−0.155	**−0.691**	0.020	‒0.235	−0.318	0.056
	0.005	0.110	0.020	0.460	0.003	0.941	0.348	0.290	0.836
Hydroxytyrosol acetate	−0.273	0.018	−0.543	0.122	−0.566	−0.299	‒0.335	−0.308	0.253
	0.001	0.950	0.030	0.562	0.022	0.244	0.174	0.306	0.345
DOA	0.065	**0.768**	−0.490	0.251	0.086	**0.685**	0.130	−0.385	0.533
	0.454	0.001	0.054	0.226	0.751	0.002	0.607	0.194	0.034
DLA	0.055	0.568	−0.344	0.341	0.020	0.323	0.260	−0.478	**0.798**
	0.526	0.027	0.192	0.095	0.942	0.206	0.297	0.099	<0.001
Lignans + Cinnamic ac.	−0.210	0.071	−0.112	0.431	0.063	−0.269	0.016	0.287	0.216
	0.014	0.802	0.680	0.032	0.816	0.297	0.948	0.342	0.422
AOA	0.272	0.559	−0.284	0.096	0.268	0.293	0.008	−0.127	0.449
	0.001	0.030	0.287	0.649	0.315	0.254	0.975	0.680	0.081
ALA	0.239	−0.376	−0.223	0.049	0.197	0.462	−0.121	−0.167	0.381
	0.005	0.167	0.406	0.816	0.465	0.062	0.632	0.585	0.145
Ferulic acid	−0.234	**−0.624**		−0.117	**−0.667**	−0.290	−0.267	0.018	−0.123
	0.006	0.013		0.577	0.005	0.258	0.283	0.953	0.649
Luteolin	−0.156	−0.280	**−0.601**	−0.040	**−0.816**	−0.140	−0.272	−0.216	0.207
	0.071	0.312	0.014	0.848	<0.001	0.592	0.274	0.478	0.441
Apigenin	−0.155	−0.364	−0.533	−0.080	**−0.823**	−0.119	0.086	−0.563	0.555
	0.071	0.182	0.033	0.705	<0.001	0.649	0.734	0.045	0.026
Total phenols	0.137	**0.634**	−0.434	0.259	0.141	0.533	0.085	−0.296	**0.670**
	0.112	0.011	0.093	0.212	0.603	0.027	0.739	0.326	0.004
Secoiridoid derivatives	0.162	**0.672**	−0.436	0.257	0.171	0.546	0.095	−0.270	**0.669**
	0.059	0.006	0.091	0.215	0.526	0.023	0.707	0.373	0.005
*o*-Diphenols	0.158	**0.706**	−0.496	0.214	0.169	**0.636**	0.068	−0.270	0.540
	0.067	0.003	0.051	0.304	0.530	0.006	0.789	0.373	0.031

^1^ Pearson coefficient; ^2^ Significance expressed by *p*-value.

**Table 5:** Standardized canonical discriminant function coefficients.

Parameter	Function						
	1	2	3	4	5	6	7
Palmitoleic	0.446	0.188	−0.394	−0.159	0.242	−0.307	−0.046
Margaric	0.456	0.825	0.160	0.121	−0.146	−0.013	0.098
Linoleic	−0.840	0.378	−0.275	0.386	0.242	−0.011	−0.042
Arachidic	−0.457	0.173	0.456	−0.597	−0.466	−0.209	0.091
Gadoleic	0.274	0.167	−0.046	0.771	0.080	0.597	0.115
Hydroxytyrosol	0.016	0.188	−1.049	−1.000	−0.285	2.042	0.734
Tyrosol	0.151	−0.569	1.389	1.263	0.230	−1.735	−0.931
*p*-Coumaric ac.	−0.181	0.101	0.745	−0.156	0.392	0.362	−0.470
Hydroxytyrosol acetate	0.439	−0.186	−0.300	−0.084	0.377	0.018	0.415
DOA	0.606	−0.184	0.529	−0.497	0.866	−0.468	−0.329
DLA	−0.592	0.120	−0.457	0.737	−0.304	−0.086	0.892
Lignans + cinnamic ac.	−0.252	0.320	−0.662	−0.489	−0.463	−0.087	−0.412

**Table 6:** Results of the stepwise linear discriminant analysis for all the cultivars.

Cultivar	Predicted Group Membership (Number of Samples and Correct Classification Percentage)								Total
	Arbequina	Cornicabra	Manzanilla Cacereña	Manzanilla de Sevilla	Morisca	Pico Limón	Picual	Verdial de Badajoz	
Arbequina	15 (100%)	0	0	0	0	0	0	0	15 (100%)
Cornicabra	0	14 (87.5%)	0	0	1 (6.3%)	0	1 (6.3%)	0	16 (100%)
Manzanilla Cacereña	0	0	22 (91.7%)	1 (4.2%)	1 (4.2%)	0	0	0	24 (100%)
Manzanilla de Sevilla	0	0	0	16 (100%)	0	0	0	0	16 (100%)
Morisca	0	0	0	0	17 (100%)	0	0	0	17 (100%)
Pico Limón	0	0	0	0	1 (5.6%)	17 (94.4%)	0	0	18 (100%)
Picual	0	0	0	0	0	0	13 (100%)	0	13 (100%)
Verdial de Badajoz	0	0	0	0	0	0	0	15 (100%)	15 (100%)

## Abbreviations

ALA	aldehydic form of ligstroside aglycone
AOA	aldehydic form of oleuropein aglycone
DLA	decarboxymethyl form of ligstroside aglycone
DOA	decarboxymethyl form of oleuropein aglycone
EVOO	extra virgin olive oil; FA: fatty acid
MUFA	monounsaturated fatty acid
OOs	olive oils; OS: oxidative stability
PUFA	polyunsaturated fatty acid
SFA	saturated fatty acid
UFA	unsaturated fatty acid
VOO	virgin olive oil

## References

[1] Keys A., Menotti A., Karvonen M.J., Aravanis C., Blackburn H., Buzina R., Djordjevic B.S., Dontas A.S., Fidanza F., Keys M.H. (1986). The diet and 15 year death rate in the Seven Countries Study. Am. J. Epidemiol..

[2] Obied H.K., Prenzler P.D., Omar S.H. (2012). Pharmacology of olive biophenols. Adv. Mol. Toxicol..

[3] Servili M. (2014). The phenolic compounds: A comercial argument in the economic war to come on the quality of olive oil?. Oilseed Fats Crops Lipids.

[4] Mateos R. (2002). Caracterización de componentes fenolicos del aceite de oliva y su relación con la estabilidad oxidativa y el amargor. Ph.D. Thesis.

[5] Frankel E.N. (1985). Chemistry of free radical and singlet oxidation of lipids. Prog. Lipid Res..

[6] Paiva-Martins F., Gordon M.H. (2005). Interactions of ferric ion with olive oil phenolic compounds. J. Agric. Food Chem..

[7] Aparicio R., Roda L., Albi A., Gutiérrez F. (1999). Effect of various compounds on virgin olive oil stability measured by Rancimat. J. Agric. Food Chem..

[8] Gutiérrez-Rosales F., Jiménez B., Ruíz A., Albi M.A. (1999). Effect of olive ripeness on the stability of virgin olive oil extracted from the varieties Picual and Hojiblanca and on the different components involved. J. Agric. Food Chem..

[9] Rotondi A., Bendini A., Cerretani L., Mari M., Lercker G., Gallina T. (2004). Effect of olive ripening degree on the oxidative stability and organoleptic properties of cv. Nostrana di Brisighella extra virgin olive oil. J. Agric. Food Chem..

[10] Mateos R., Trujillo M., Pérez-Camino M.C., Moreda W., Cert A. (2005). Relationship between oxidative stability, triacylglicerol composition, and antioxidant content in olive oil matrices. J. Agric. Food Chem..

[11] Bendini A., Cerretani L., Carrasco-Pancorbo A., Gómez-Caravaca A.M., Segura-Carretero A., Fernández-Gutiérrez A., Lercker G. (2007). Phenolic molecules in virgin olive oils: A survey of their sensory properties, health effects, antioxidant activity and analytical methods. An overview of the last decade. Molecules.

[12] Briante R., Febbraio F., Nucci R. (2003). Antioxidant properties of low molecular weight phenols present in the Mediterranean diet. J. Agric. Food Chem..

[13] Frankel E.N. (1996). Antioxidants in lipid food and their impact on food quality. Food Chem..

[14] Tous J., Uceda M., Romero A., Beltrán G., Días I., Jiménez A., Rallo L., Barranco D., Caballero J.M., del Río C., Martín A., Tous J., Trujillo I. (2005). Composición del Aceite. En: Variedades de Olivo en España (Libro II: Variabilidad y Selección).

[15] Rondanini D.P., Castro D.N., Searles P.S., Rousseaux M.C. (2011). Fatty acid profiles of varietal virgin olive oils (*Olea europea* L.) from mature orchards in warm arid valleys of Norhtwestern Argentina (La Rioja). Grasas y Aceites.

[16] Talhaoui N., Gómez-Caravaca A.M., León L., de la Rosa R., Fernández-Gutiérrez A., Segura-Carretero A. (2016). From olive fruits to olive oil: Phenolic compound transfer in six different olive cultivars grown under the same agronomical conditions. Int. J. Mol. Sci..

[17] Andjelkovic M., van-Camp J., Pedra M., Renders K., Socaciu C., Verhé R. (2008). Correlations of the phenolic compounds and the phenolic content in some spanish and french olive oils. J. Agric. Food Chem..

[18] Ramos-Escudero F., Morales M.T., Asuero A.G. (2015). Characterization of bioactive compounds from monovarietal virgin olive oil: Relationship between phenolic compounds-antioxidant capacities. Int. J. Food Prop..

[19] Beltrán G., Ruano M.Y., Jiménez A., Uceda M., Aguilera M.P. (2007). Evaluation of virgin olive oil bitterness by total phenol content analysis. Eur. J. Lipid Sci. Technol..

[20] Fuentes de Mendoza M. (2013). Caracterización de los Aceites de Oliva Virgen producidos en la zona oleícola de Tierra de Barros. Ph.D. Thesis.

[21] Jerman-Klen T., Golc-Wondra A., Vrhsovsek U., Sivilotti P., Mozetic B. (2015). Olive fruit phenols transfer, transformation, and partition trail during laboratory-scale olive oil processing. J. Agric. Food Chem..

[22] Gracia M.S., Royo A., Guillén M. (2009). Composición química de aceites de las variedades Arbequina y Empletre cultivadas en regadío. Grasas Aceites.

[23] Romero-Aroca A. (2011). Caracterización y Diferenciación de los Aceites Vírgenes de Oliva de la Comarca del Priorat (Tarragona), dentro del Mercado Global de Aceites de la Variedad “Arbequina”. Ph.D. Thesis.

[24] Yousfi K., Weiland C.M., García J.M. (2012). Effect of harvesting system and fruit cold storage on virgin olive oil chemical composition and quality of superintensive cultivated “Arbequina” olives. J. Agric. Food Chem..

[25] Pardo J.E., Sena E., Cuesta M.A., Granell J.D., Valiente J., Alvarez-Ortí M. (2013). Evaluation of potential and real quality of virgin olive oil from “Campos de Hellín” (Albacete, Spain). J. Am. Oil Chem. Soc..

[26] Mattar S., Turcato A. (2006). Correlaciones entre los parámetros químicos y sensoriales del aceite de oliva virgen de San Juan. Aceites y Grasas.

[27] Torres M.M., Pierantozzi P., Cáceres M.E., Labombarda P., Fontanazza G., Maestri D.M. (2009). Genetic a chemical assessment of “Arbequina” olive cultivar grown in Córdoba province, Argentina. J. Sci. Food Agric..

[28] El Antari A., El Moudni A., Ajana H. (2003). Comparación de la calidad y la composición acidica del aceite de olive de algunas variedades mediterráneas cultivadas en Marruecos. Olivae.

[29] Berenguer M.J., Vossen P.M., Grattan S.R., Connell J.H., Polito V.S. (2006). Tree irrigation levels for optimum chemical and sensory properties of olive oil. Hortic. Sci..

[30] Gómez-Rico A., Salvador M.D., Moriana A., Pérez D., Olmedilla N., Ribas F., Fregapane G. (2007). Influence of different irrigation strategies in a traditional Cornicabra cv. Olive orchard on virgin olive oil composition and quality. Food Chem..

[31] Morales-Sillero A., García J.M. (2015). Impact assessment of mechanical harvest on fruit physiology and consequences on oil physicochemical and sensorial quality from “Manzanilla de Sevilla” and “Manzanilla Cacereña” super-high density hedgerows. A preliminary study. J. Sci. Food Agric..

[32] Sánchez-Casas J., de Miguel Gordillo C., Osorio Bueno E., Marín Expósito J., Gallardo González L., Martínez Cano M. (2006). Calidad sensorial de aceites de oliva virgen procedentes de variedades de aceitunas producidas en Extremadura. Grasas y Aceites..

[33] Sánchez de Medina V., Priego-Capote F., Luque de Castro M.D. (2015). Characterization of monovarietal virgin olive oils by phenol profiling. Talanta.

[34] De la Rosa R., Talhaoui N., Rouis H., Velasco L., León L. (2013). Fruit characteristics and fatty acid composition in advanced olive breeding selections along the ripening period. Food Res. Int..

[35] Sánchez-Casas J. (2015). Caracterización del aceite procedente de las principales variedades de aceitunas de Extremadura. Ph.D. Thesis.

[36] Romero M.P., Motilva M.J., Preedy V.R., Watson R.R. (2010). Effect of climatic conditions on quality of virgin olive oil—Chapter 5. Olives and Olive Oil in Health and Disease Prevention.

[37] Allalout A., Krichene D., Methenni K., Taamalli A., Daoud D., Zarrouk M. (2011). Behavior of super-intensive Spanish and Greek olive cultivars grown in northern Tunisia. J. Food Biochem..

[38] Dag A., Kerem Z., Yogev N., Zipori I., Lavee S., Ben-David E. (2011). Influence of time of harvest and maturity index on olive oil yield and quality. Sci. Hortic..

[39] García-Inza G.P., Castro D.N., Hall A.J., Rousseaux M.C. (2014). Responses to temperature of fruit dry weight, oil concentration, and oil fatty acid composition in olive (Olea europea L. var. “Arauco”). Eur. J. Agron..

[40] Sánchez-Casas J., Osorio Buenos E., Montaño A.M., Martínez Cano M. (2003). Estudio del contenido en ácidos grasos de aceites monovarietales elaborados a partir de aceituna producidas en la región extremeña. Grasas y Aceites.

[41] Vossen P. (2009). Olive Cultivar Comparisons from Around the World.

[42] Reboredo-Rodríguez P., González-Barreiro C., Cancho-Grande B., Fregapane G., Salvador M.D., Simai-Gándara J. (2015). Characterization of extra virgin olive oils from Galician autochthonous varieties and their co-crushing with Arbequina and Picual cv.. Food Chem..

[43] Reboredo-Rodríguez P., González-Barreiro C., Cancho-Grande B., Fregapane G., Salvador M.D., Simai-Gándara J. (2015). Blending Local olive oils with Arbequina or Picual oils produces high quality, distinctive EVOOs. Eur. J. Lipid Sci. Technol..

[44] Aranda F., Gómez-Alonso C., Rivera del Álamo R.M., Salvador M.D., Fregapane G. (2004). Triglyceride, total and 2-position fatty acid composition of Cornicabra virgin olive oil: Comparison with other Spanish cultivars. Food Chem..

[45] Salvador M.D., Aranda F., Gómez-Alonso S., Fregapane G. (1999). Contribution of chemical components of Cornicabra virgin olive oils to oxidative stability. A study of three successive crop seasons. J. Am. Oil Chem. Soc..

[46] Salvador M.D., Aranda F., Gómez-Alonso S., Fregapane G. (2001). Influence of fruit ripening on Cornicabra virgin olive oil. A study of four crop seasons. Food Chem..

[47] Aguilera M.P., Beltrán G., Ortega D., Fernández A., Jiménez A., Uceda M. (2005). Characterization of virgin olive oil of Italian olive cultivar: “Frantoio” and “Lecciono”, grown in Andalusia. Food Chem..

[48] Ouni Y., Taamlli A., Guerfel M., Abdelly C., Zarrouk M., Flamini G. (2012). The phenolic compounds and compositional quality of Chétoui virgin olive oil: Effect of altitude. Afr. J. Biotechnol..

[49] Uceda M., Aguilera M.P., Jiménez A., Beltrán G., Gutiérrez A.F., Carretero A.S. (2009). Chapter 4: Variedades de olivo y aceituna. Tipos de Aceites. El Aceite de Oliva Virgen: Tesoro de Andalucía. 13 perspectivas concatenadas.

[50] Gomez-Rico A., Salvador D., Fregapanne G. (2009). Virgin olive oil fruit minor constituents as affected by irrigation management base don SWP and TDF as compared to ETc in medium-density young olive orchards (*Olea europaea* cv. Cornicabra and Morisca). Food Res. Int..

[51] Luaces P., Romero C., Gutiérrez F., Sanz C., Pérez A.G. (2007). Contribution of olive seed to the phenolic profile and related quality parameters of virgin olive oil. J. Sci. Food Agric..

[52] Uceda M., Beltrán G., Jiménez A., Rallo L., Barranco D., Caballero J.M., del Río C., Martín A., Tous J., Trujillo I. (2005). Composición del aceite (Banco de germoplasma Mundial de Córdoba).

[53] Martínez J.M., Muñoz-Aranda E., Alba J., Lanzón A. (1975). Informe sobre la utilización del analizador de rendimientos Abencor. Grasas y Aceites.

[54] Mateos R., Cert A., Pérez-Camino M.C., García J.M. (2004). Evaluation of virgin olive bitterness by quantification of secoiridoid derivates. J. Am. Oil Chem. Soc..

[55] Mateos R., Espartero J.L., Trujillo M., Rios J.J., León-Camacho M., Alcudia F. (2001). Determination of phenols, flavones and lignans in virgin olive oils by solid-phase extraction and high performance liquid chromatography with diode array ultraviolet detection. J. Agric. Food Chem..

[56] European Union Commission (1991). Regulation EEC 2568/91 on the characteristics of olive oil and olive pomace and their analytical methods. Off. J. Eur. Communities.

[57] Gutiérrez F. (1989). Determinación de la estabilidad oxidativa de aceites de oliva vírgenes. Comparación entre el método del oxígeno activo (OAM) y el método Rancimat. Grasas y Aceites.

